# Virtual daily living test to screen for mild cognitive impairment using kinematic movement analysis

**DOI:** 10.1371/journal.pone.0181883

**Published:** 2017-07-24

**Authors:** Kyoungwon Seo, Jae-kwan Kim, Dong Hoon Oh, Hokyoung Ryu, Hojin Choi

**Affiliations:** 1 Department of Industrial Engineering, Hanyang University, Seoul, Republic of Korea; 2 Department of Arts & Technology, Hanyang University, Seoul, Republic of Korea; 3 Institute for Health and Society, Hanyang University, Seoul, Republic of Korea; 4 Seulha Mental Health Clinic, Jeju, Republic of Korea; 5 Department of Neurology, College of Medicine, Hanyang University, Seoul, Republic of Korea; Banner Alzheimer's Institute, UNITED STATES

## Abstract

Questionnaires or computer-based tests for assessing activities of daily living are well-known approaches to screen for mild cognitive impairment (MCI). However, questionnaires are subjective and computerized tests only collect simple performance data with conventional input devices such as a mouse and keyboard. This study explored the validity and discriminative power of a *virtual daily living test* as a new diagnostic approach to assess MCI. Twenty-two healthy controls and 20 patients with MCI were recruited. The virtual daily living test presents two complex daily living tasks in an immersive virtual reality environment. The tasks were conducted based on subject body movements and detailed behavioral data (i.e., kinematic measures) were collected. Performance in both the proposed virtual daily living test and conventional neuropsychological tests for patients with MCI was compared to healthy controls. Kinematic measures considered in this study, such as body movement trajectory, time to completion, and speed, classified patients with MCI from healthy controls, *F*(8, 33) = 5.648, *p* < 0.001, η^2^ = 0.578. When both hand and head speed were employed in conjunction with the immediate free-recall test, a conventional neuropsychological test, the discrimination power for screening MCI was significantly improved to 90% sensitivity and 95.5% specificity (cf. the immediate free-recall test alone has 80% sensitivity and 77.3% specificity). Inclusion of the kinematic measures in screening for MCI significantly improved the classification of patients with MCI compared to the healthy control group, Wilks’ Lambda = 0.451, *p* < 0.001.

## Introduction

Mild cognitive impairment (MCI) is an intermediate clinical stage between normal aging and dementia. The main characteristics of MCI [1,2] are as follows: i) absence of dementia, ii) normal general cognitive function, iii) intact activities of daily living, iv) concern regarding a change in cognition, and v) objective evidence of impairment in one or more cognitive functions. It has been reported that patients with MCI have a higher rate of progression to dementia than cognitively normal individuals [3]. Because of this increased risk, early screening and ensuing interventions are important for patients with MCI [4].

Prior to the development of MCI, it is important to carefully assess a patient’s instrumental activities of daily living (IADL), such as housework, shopping, and daily medication [5]. IADL tasks are all related to a patient’s cognitive functioning for daily living based on memory, executive function, language, and psychomotor speed [6]. A systematic review by Gold [7] showed that the most cognitively demanding IADL tasks, such as handling finances and using public transportation could be used to efficiently screen MCI patients.

Although the questionnaire-based IADL assessment is still being used [8], recent studies have pointed out that there is a considerable discrepancy between the outcomes of recall-based questionnaires and actual IADL performance. For instance, many patients over- or under-estimate their capacities when responding to IADL questions irrespective of their actual IADL performance [9]. When more cognitively complex IADL tasks are given, this discrepancy increases [10]. Therefore, the questionnaire-based IADL assessment is limited in its ability to sensitively screen MCI patients in clinical settings [11].

Recently, virtual reality (VR) technology has been applied to directly measure IADL task performance in the hope that behavioral results while performing VR tasks (e.g., time to completion or the number of errors) will mimic actual IADL performance. For instance, Allain et al. [12] demonstrated that both virtual and real coffee-making tasks were highly correlated in terms of behavioral results. Klinger et al. [13] used a non-immersive VR environment for mailing and shopping tasks to determine the relationship between VR task completion time and neuropsychological tests and found that these two measures highly correlated with each other. Note that previous VR studies on IADL task performance measured only simple performance data such as reaction time with conventional input devices such as a mouse and keyboard. In this regard, Jekel et al. [14] proposed that detailed performance analysis of the behavioral results is needed to efficiently screen for MCI, but there has not yet been an empirical study to examine this proposal.

Kinematic analysis of body movements is a potential solution to analyze detailed IADL task performance. For example, Schröter et al. [15] performed kinematic analysis of handwriting movements in terms of the frequency of hand-shaking, the number of changes in direction, and mean peak velocity, and found that MCI patients had abnormal hand motor function. Montero-Odasso et al. [16] studied gait analysis (i.e., gait velocity, step length, stride length, step time, stride time, and double support time) in MCI patients and identified significant connections between their walking patterns and cognitive function. After reviewing kinematic studies about daily living tasks, de los Reyes-Guzmán and colleagues [17] proposed representative kinematic measures of body movement including trajectory, time to completion, and speed. These kinematic measures could provide the opportunity to quickly assess a patient’s cognitive impairment [18], and we believe that the body movement kinematic measures while naturally performing VR-based IADL tasks would sensitively detect MCI compared to unnatural interaction with a mouse and keyboard.

This study thus developed an immersive *virtual daily living test (VDLT)* that asked both MCI patients and healthy controls to naturally perform complex IADL tasks in an immersive virtual environment, and analyzed their body movements to determine the most sensitive kinematic measures for screening patients with MCI. The two complex IADL tasks used for the VDLT were “Task 1: Withdraw money” (handling a finance task) and “Task 2: Take a bus” (using public transportation task). The VDLT collected kinematic (e.g., body movement trajectory, time to completion, and speed) and behavioral data (e.g., number of errors) while conducting the two IADL tasks. The body movement trajectory is the total moving distance of hand and/or head motion, and the time to completion means how long it took to complete each task. The movement speed is calculated from the body movement trajectory divided by the time to completion. Note that the kinematic measures are defined in de los Reyes-Guzmán et al.’s study [17]. The kinematic data revealed subtle functional changes in performing instrumental activities, which can be of value for identifying dementia or MCI [19].

The current empirical study is significant for three reasons. First, we explored the clinical potential of VDLT by comparing kinematic measures (i.e., hand/head movement trajectory, time to completion, and speed) between MCI patients and age-matched healthy controls in a VDLT environment. Second, the validity of VDLT was demonstrated through the correlation analysis between VDLT and conventional neuropsychological test results. Finally, the enhanced discriminative power of VDLT for screening MCI patients was confirmed by discriminant analysis.

## Materials and methods

### Participants

This study utilized a case-control design. We recruited 22 healthy controls (14 males and eight females) and 20 MCI patients (12 males and eight females) from a tertiary medical center, Hanyang University Hospital. The individual in this manuscript has given written informed consent (as outline in PLOS consent form) to publish these case details. Written informed consent form was obtained from each subject after the experimental procedure was explained. This study was approved by the Institutional Review Board of the Hanyang University according to the Declaration of Helsinki (HYI-15-029-2).

Healthy controls were recruited from a pool of community volunteers with no reported health problems in the medical center. MCI patients were randomly selected from outpatients in the Department of Neurology at Hanyang University Hospital, and voluntarily participated in the study. MCI was diagnosed with criteria described by Albert et al. [20]. Neuropsychological tests, physical examination, and medical history review were completed by a neurologist with 17 years of experience. The subjects who abused drugs or drank alcohol heavily within four weeks of starting the study were excluded through clinical interview by the neurologist. Other exclusion criteria were a history of neurological/psychiatric diseases and brain surgery.

### Neuropsychological tests

A total of seven neuropsychological tests were administered to the enrolled subjects. These included: i) Mini mental state examination-dementia screening (MMSE-DS) for assessing general cognitive function [21]; ii) Korean instrumental activities of daily living (K-IADL) for assessing IADL deficits [22]; iii) free and cued selective reminding test (FCSRT) for immediate and delayed free-recall memory [23]; iv) digit span test-forward (DST-F); v) digit span test-backward (DST-B) for executive function [24]; vi) trail making test-A (TMT-A), and vii) trail making test-B (TMT-B) for psychomotor speed [24].

### Virtual daily living test (VDLT)

The VDLT was an immersive VR-based test that measured a subject’s body movements while performing IADL tasks. The experimental setting for the VDLT was a room-sized cube (4m x 2.5m x 2.5m) that had four rear-projection screens which could project an immersive and realistic 3D environment. Subjects wore stereoscopic glasses (weighs around 50g) and reflective markers (weighs less than 1g) on dominant hand and head. The VDLT consisted of two IADL tasks: “Task 1: Withdraw money” and “Task 2: Take a bus.” While performing the two tasks, eight motion tracking cameras (OptiTrack^™^, NaturalPoint Inc., USA) recorded the subject’s body movements by tracking the markers.

**Fig 1:**
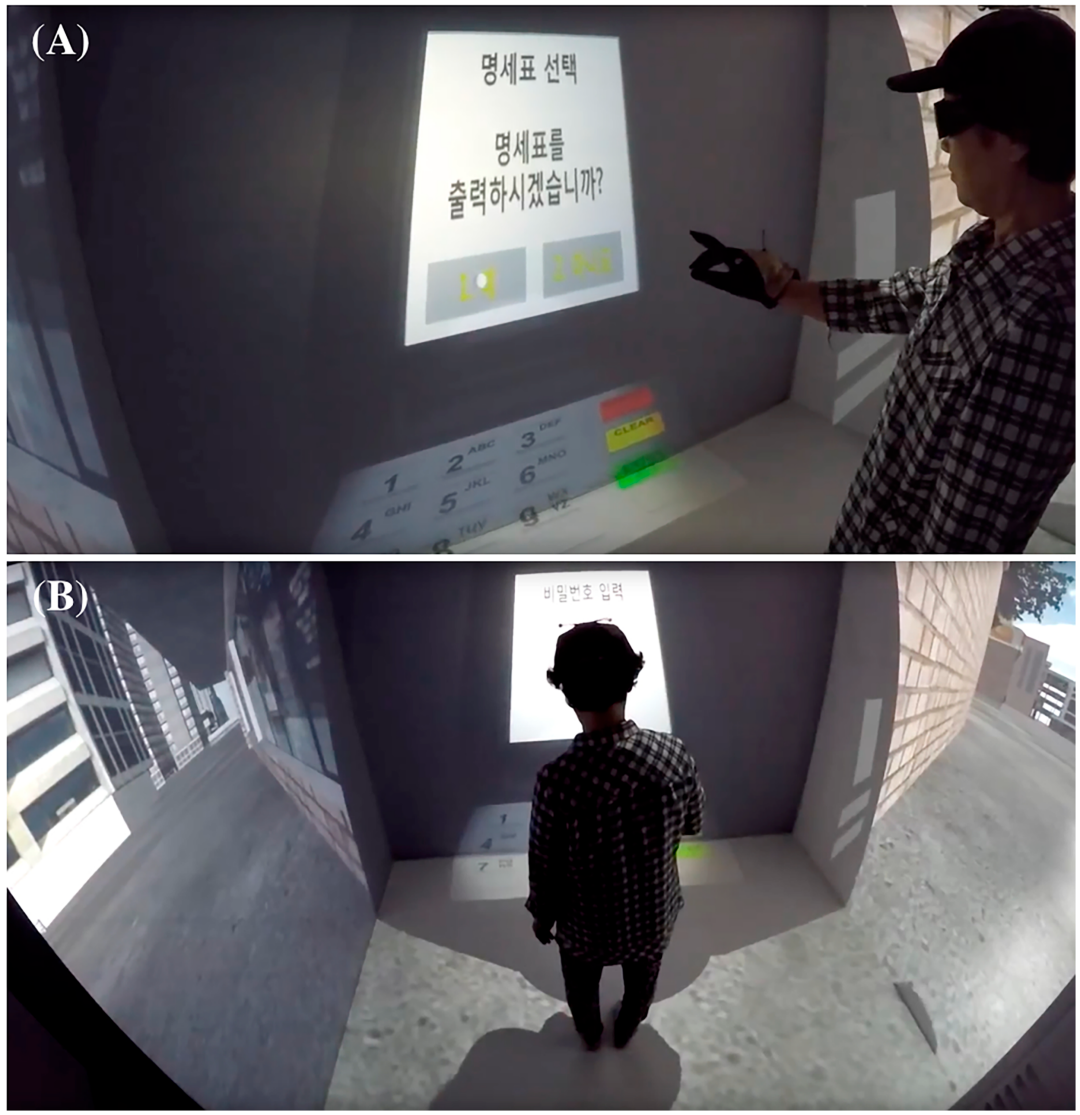
“Task 1: Withdraw money” in the virtual daily living test. (A) The front view of subject. (B) The back view while conducting the test.

“Task 1: Withdraw money” (see Fig 1) was carried out by eight action steps: i) insert the cash-card; ii) select the menu to withdraw money; iii) select the amount to withdraw; iv) select the note types to match the amount; v) enter the password of the account; vi) select the option for receipt; vii) remove the cash-card; and viii) take out the money. Prior to the experiment, the following instructions for Task 1 performance were given in the same manner to all subjects: “Please withdraw 70,000 KRW (equivalent to around 60 USD) from the ATM for shopping. You should select two different note types (a note for 50,000 KRW and two notes for 20,000 KRW). The password for the ATM was automatically set to today’s date (e.g., when the experiment was carried out on the 11^th^ of November = 1111; note that this was intentional to equate with one of the MMSE-DS questions, “What is today’s date?”). Be sure to keep the receipt.” While the subjects conducted the task, the eight motion tracking cameras collected the position of both dominant hand and head movement in a 3-dimensional Cartesian coordinate system. This collection of raw coordinate data was converted into three kinematic measures. The total distance of dominant hand movement calculated by summing distances between sequential hand positions while performing the task (i.e., hand trajectory), the time taken to finish the whole task (i.e., time to completion), and the mean velocity of the dominant hand during the task (i.e., hand speed). In addition, the number of errors, such as withdrawing a different amount of money, selecting incorrect note types, entering the wrong password (i.e., the date of the experiment happened), and forgetting to keep the receipt, were collected as behavioral data.

**Fig 2:**
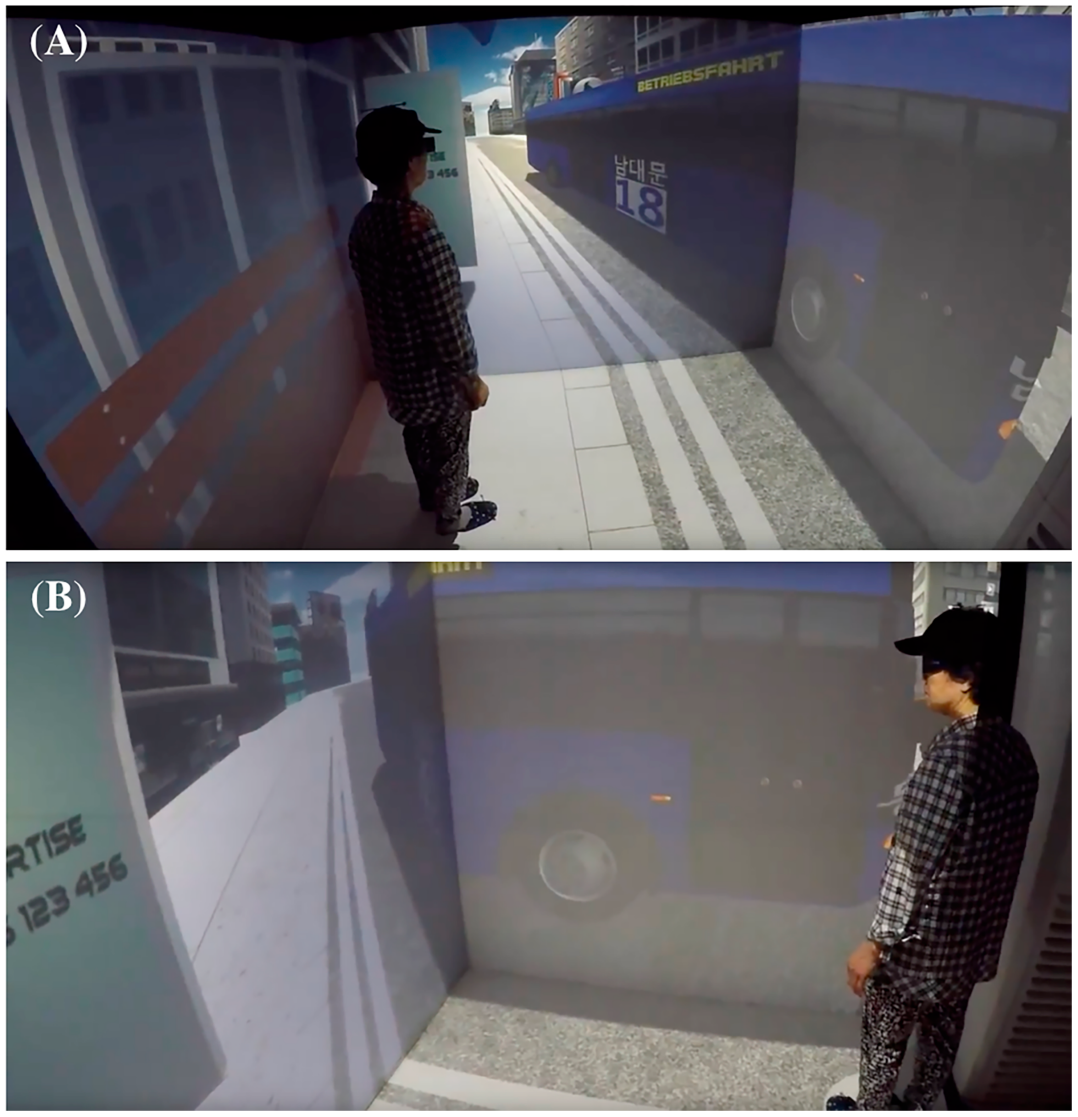
“Task 2: Take a bus” in the virtual daily living test. (A) Subject waits at a bus stop. (B) Subject steps into the door of the bus when the target bus arrives.

“Task 2: Take a bus” required the subject to wait at a bus stop and get on the correct bus to reach their destination (see Fig 2). The target bus could be identified by the number of the bus line, the color of the bus, and the destination presented on the bus. To test the subjects, incorrect buses which had different numbers, colors, and destinations also randomly arrived at the bus stop during the task. When the target bus arrived, the subject had to walk out of the bus stop and step into the door of the bus. Each subject repeated this task eight times with different target buses. The target bus information in each trial was shown in the VR environment until subjects confirmed the target bus information. While conducting the task, kinematic measures were collected as follows: the total distance of head movement during the task (i.e., head trajectory), the time taken to finish the whole task (i.e., time to completion), and the mean velocity of the head while taking a bus (i.e., head speed). The number of errors, such as taking incorrect buses or missing the correct bus, were additionally collected as part of the behavioral data.

### Procedure

All the experiments (i.e., neuropsychological tests and VDLT) were supervised by a specially trained psychologist on a one-to-one basis. The order of neuropsychological tests and VDLT was counter-balanced. Prior to the VDLT, all subjects received a 10 minute training session to become accustomed to the immersive virtual environment. Only two subjects were asked to do an extra training session, given by the psychologist’s judgment. Subjects then independently performed the two VDLT tasks, which were also randomly ordered.

### Statistical analyses

To examine the validity and discriminative power of the VDLT as a diagnostic tool for MCI screening, several statistical methods were applied using IBM SPSS Statistics 21. First, prior to the main statistical analyses, a chi-square test and multivariate analysis of variance (MANOVA) examined the group differences in basic demographic characteristics and neuropsychological test results. Second, MANOVA was again used to investigate the group differences in the VDLT results. For all MANOVAs, a Bonferroni-adjusted alpha level was used for multiple comparisons and effect sizes were reported by partial eta squared (η^2^). Third, a Pearson correlation analysis for validity check was conducted between the neuropsychological tests and VDLT. Finally, a forward stepwise linear discriminant analysis (LDA) was carried out to identify the discriminative power of the VDLT in order to screen patients for MCI.

## Results

### Basic demographic characteristics and neuropsychological test results

A chi-square test revealed no significant effect of gender on each subject group, χ^2^(1) = 0.059, *p* = 0.808. In addition, to check for demographic and neuropsychological differences, a one-way MANOVA (Table 1), in which demographic characteristics (i.e., age, education level, GDSSF-K) and neuropsychological test results were treated as dependent variables (i.e., 12 dependent variables), and subject group (i.e., healthy controls vs. MCI patients) was the independent factor, revealed a statistically significant multivariate effect, *F*(12, 29) = 3.552, *p* = 0.003; η^2^ = 0.595. In the subsequent univariate analyses, using a Bonferroni-adjusted alpha level for multiple comparisons (i.e., 0.05/12 = 0.0042), it was found that there were no statistical differences in age, education level, or depression level (GDSSF-K) [25] between healthy controls and MCI patients. In the neuropsychological test results, MCI patients had statistically significantly lower scores in immediate free-recall memory (FCSRT) compared to healthy controls. As briefly discussed in the Introduction, the questionnaire-based IADL assessment (K-IADL) was not statistically different between healthy controls and MCI patients due to a ceiling effect.

**Table 1:** Basic demographic characteristics and neuropsychological test results.

	Healthy controls	MCI patients	*F*(1, 40)	*p*	η^2^
**Demographic variables**					
Number of subjects (male)	22 (14)	20 (12)	-	0.808	-
Age	72.3±3.7	72.4±3.9	0.004	0.948	<0.001
Education level	9.6±3.3	9.0±4.7	0.299	0.587	0.007
GDSSF-K	3.2±2.0	4.9±3.8	3.277	0.078	0.076
**Neuropsychological tests**					
MMSE-DS	28.0±1.6	25.9±2.7	7.940	0.007	0.166
K-IADL	10 (max: 10)	10 (max: 10)	-	-	-
FCSRT (number of immediate recall)	36.2±5.0	27.8±6.6	22.117	<0.001	0.356
FCSRT (number of delayed recall)	13.6±2.3	11.5±2.6	7.438	0.009	0.157
DST-F (number of correct answers)	9.1±1.8	8.3±2.7	1.417	0.241	0.034
DST-B (number of correct answers)	7.3±2.1	5.6±1.8	7.655	0.009	0.161
TMT-A (time to completion, seconds)	66.8±24.0	89.6±44.4	4.376	0.043	0.099
TMT-A (number of errors)	0.4±0.7	0.7±1.0	1.737	0.195	0.042
TMT-B (time to completion, seconds)	127.8±44.3	144.3±44.1	1.450	0.236	0.035
TMT-B (number of errors)	1.0±1.1	1.7±1.2	3.369	0.074	0.078

Values are means±SD. GDSSF-K: geriatric depression scale short form-Korean version; MMSE-DS: mini-mental state examination-dementia screening; K-IADL: Korean instrumental activities of daily living; FCSRT: free and cued selective reminding test; DST-F: digit span test-forward; DST-B: digit span test-backward; TMT-A: trail making test-A; and TMT-B: trail making test-B.

### Differences in VDLT results between MCI patients and healthy controls

The VDLT results (i.e., 8 dependent variables) between MCI patients and healthy controls were assessed by MANOVA (Table 2). There was a statistically significant multivariate effect in the VDLT results between healthy controls and MCI, *F*(8, 33) = 5.648, *p* < 0.001, η^2^ = 0.578. In the subsequent univariate analyses, when a Bonferroni-adjusted alpha level was adopted for multiple comparisons (i.e., 0.05/8 = 0.00625), the MCI patients showed significantly slower dominant hand speed while conducting Task 1; a slower head speed and a higher number of errors in Task 2.

**Table 2:** Kinematic (trajectory, time to completion, speed) and behavioral results (number of errors) from virtual daily living test.

	Healthy controls	MCI patients	*F*(1, 40)	*p*	η^2^
**Task 1: Withdraw money**					
Hand trajectory (meters)	49.5±40.5	32.2±20.3	2.970	0.093	0.069
Time to completion (seconds)	81.1±26.5	106.5±38.0	6.417	0.015	0.138
Hand speed (m/s)	0.6±0.4	0.3±0.1	12.694	0.001	0.241
Number of errors	0.2±0.5	0.7±0.8	5.154	0.029	0.114
**Task 2: Take a bus**					
Head trajectory (meters)	125.4±35.8	100.7±24.5	6.678	0.014	0.143
Time to completion (minutes)	13.5±0.7	13.5±0.7	0.003	0.953	<0.001
Head speed (m/s)	0.4±0.1	0.3±0.1	11.029	0.002	0.216
Number of errors	0.8±0.9	2.3±1.4	16.444	<0.001	0.291

Values are means±SD.

### Correlation between the neuropsychological test and VDLT results

A Pearson correlation analysis was run to determine the validity of VDLT results (i.e., total 8 variables) against the most discriminating neuropsychological measure from this study (i.e., FCSRT immediate free-recall). A Bonferroni-adjusted alpha level was applied for multiple correlation tests (i.e., 0.05/8 = 0.00625). In Task 1, the time to completion in VDLT showed a negative moderate correlation with the immediate free-recall test, r = -0.417, *p* = 0.006. However, one of our kinematic measures, dominant hand speed did not correlate with the neuropsychological test result, which potentially provides additional power to screen MCI patients against healthy controls (note that Table 2 shows that the hand speed can separate MCI patients and healthy controls). Similarly, in VDLT Task 2, the number of errors demonstrated a negative moderate relationship with the immediate free-recall test, r = -0.417, *p* = 0.006. Head speed, a proposed kinematic measure in this study, did not correlate with the neuropsychological test result. However, as shown in Table 2, head speed did uniquely discriminate MCI patients from healthy controls.

### Discriminating performance of the kinematic measures

The results of MANOVA showed that a neuropsychological test (i.e., FCSRT immediate free-recall) and two kinematic measures (i.e., dominant hand speed and head speed) were able to differentiate between MCI patients and healthy controls. A forward stepwise LDA was applied to determine the discriminative power of the three items (i.e., FCSRT immediate free-recall, dominant hand speed, and head speed) to identify MCI patients. The stepwise LDA begins with no predictor variables and, in turn, adds the most correlated predictor which significantly decreases the ratio of within-groups sums of squares to the total sums of squares (i.e., Wilks’ Lambda). A statistically smaller Wilks’ Lambda (with an alpha level of 0.05) indicates the added predictor enhances the predictive power to the discriminant. All the variables are added until the change in Wilks’ Lambda is not significant [26].

As shown in Table 3, both a neuropsychological test (i.e., FCSRT immediate free-recall) and two kinematic measures (i.e., hand speed and head speed from the VDLT) best discriminated between MCI patients and healthy controls (accuracy: 92.9%; sensitivity: 90%; and specificity: 95.5%). In effect, hand and head speed, both of which did not correlate with the neuropsychological test score, significantly contributed to improved sensitivity and specificity for discriminating MCI subjects from healthy controls.

**Table 3:** Discriminant analysis for classifying the two diagnostic groups.

Steps	Predictor variables	Wilks’ Lambda (*p*)	Accuracy (%)	Sensitivity (%)	Specificity (%)
**Step 1**	FCSRT immediate free-recall	0.644 (< 0.001)	78.6	80.0	77.3
**Step 2**	FCSRT immediate free-recall Hand speed	0.512 (< 0.001)	85.7	85.0	86.4
**Step 3**	FCSRT immediate free-recall Hand speed Head speed	0.451 (< 0.001)	92.9	90.0	95.5

## Discussion

The present study was designed to apply kinematic analysis of IADL tasks in an immersive VR environment to efficiently screen for MCI. The VDLT tasks proposed in this study have two significant distinctions compared to those in previous studies. First, the VDLT was carried out by giving visual and auditory information in the immersive VR simulation. This could potentially be employed for further training [27], rehabilitation [28], and to determine the effect of therapy [29]. Second, the VDLT can be used for evidence-based clinical decision-making by quantitatively analyzing body movements [30]. These two tasks would benefit clinicians by allowing them to examine a patient’s status with more realistic daily living performance measures. To our knowledge, this is the first study of its kind to apply kinematic measures to screen for MCI patients using an immersive VR environment.

The main objectives of this study were to determine the validity and discriminative power of VDLT as a new diagnostic approach to assess MCI. The VDLT can provide observable and quantifiable data from a subject’s whole-body movement while performing complex IADL tasks in real-time. The fact that the correlation analyses with the conventional neuropsychological test was moderate means the VDLT has an additional discriminative value. The kinematic measures identified in this study, dominant hand and head speed, did not have associations with the conventional neuropsychological test result, but they can provide behavioral markers to see MCI patients against healthy controls. Note that results from the questionnaire-based K-IADL used in this study did not separate MCI patients from healthy controls. However, the VDLT sensitively differentiated MCI patients from healthy controls using virtual IADL tasks. Of course, this does not mean that other neuropsychological tests should be discarded, since our study only confirmed that kinematic measures (i.e., hand and head speed from the VDLT) in conjunction with a neuropsychological test (i.e., FCSRT immediate free-recall) best discriminated MCI patients from healthy controls. That is, the immediate free-recall test (FCSRT) had a certain benefit when used with the kinematic measures. In light of these results, kinematic data in VDLT could be a promising measure to sensitively screen patients for MCI in clinical settings.

In our opinion, the added value of the present work lies in the fact that assessment of MCI patients was made by kinematic measures using immersive VR tasks which sufficiently mimic real-world conditions. Although VDLT results seem to be a sensitive marker to assess MCI, it is important to understand to what extent VR results are different from or similar to real-world performances. Allain et al. [12] benchmarked a VR task against a closely corresponding real-world task and found that a VR paradigm could be a more complex yet ecologically valid measure. As for the reasons for the complexity of the VR, a recent study [31] proposed that VR interface with a mouse and keyboard is unnatural to use. Thus, different mental schemes would be associated in VR and real tasks, and consequently more attention will be paid to do VR tasks. Note that previous VR contents [12,13,31] were mostly based on non-immersive VR environment with a mouse and keyboard. As mentioned, the VDLT proposed in this study is an immersive VR-based test that subjects can conduct virtual IADL tasks with their own body (i.e., hand and head). In other words, subjects are free from interaction devices. It might be said that the immersive nature of our VDLT compensate the weaknesses of non-immersive VR contents by narrowing the gap between virtual and real interactions.

The present study entails a number of limitations that must be acknowledged. First, the ecological validity of VDLT compared to real-world conditions should be considered. Second, the availability of VDLT on more portable immersive VR devices like head-mounted display (e.g., Oculus Rift, HTC Vive, and etc.) would be an urgent way forward for the clinical deployment, though the difficulty of natural body movement interaction is a significant barrier to adopt such commercially-available VR systems. Third, although the VDLT used in this study discriminated MCI patients from healthy controls, clinical norm data for this modality has not been validated for use as a diagnostic tool. Fourth, we also did not determine whether other IADL tasks could be associated with the two kinematic measures (i.e., hand and head speed) identified in this study. These limitations will be further examined in the near future.

Despite these limitations, this study presented the potential of using kinematic measures from IADL tasks carried out in an immersive VR environment to correctly classify patients as MCI. This is an important finding since the VDLT overcame the limitations of questionnaire-based IADL assessment and, simultaneously, provided realistic performance data that conventional cognition-based neuropsychological tests cannot offer. These findings are also in line with other studies [32,33] that reported functional impairments are important prognostic indicators for MCI.
